# Successful treatment of severe ARDS caused by accidental inhalation of nitric acid fumes with veno-venous ECMO: A case report and literature review

**DOI:** 10.1097/MD.0000000000029447

**Published:** 2022-07-29

**Authors:** Qian Wang, Junchen Zhu, Lvlin Chen, Yan He, Hui Li, Ying Lan, Chao Huang, Liyuan Peng

**Affiliations:** Department of Critical Care Medicine, Affiliated Hospital of Chengdu University, Chengdu, China.

**Keywords:** case report, nitric acid, prone position, ultrasound, veno-venous ECMO

## Abstract

**Rationale:**

The treatment of severe acute respiratory distress syndrome caused by accidental inhalation of nitric acid fumes is challenging. Few successful cases have been reported in literature. Owing to the development of extracorporeal life support, extracorporeal membrane oxygenation (ECMO) may play an important role in treatment.

**Patient concerns:**

A 40-year-old man was accidentally exposed to nitric acid fumes for 10 minutes in a factory. Mild throat irritation and dyspnea occurred 3.5 hours after exposure. Severe dyspnea recurred approximately two hours later. Chest computed tomography revealed bilateral interstitial edema. Tracheal intubation and mechanical ventilation were provided when the non-invasive ventilator failed to support the patient. However, his vital signs, respiratory function, and circulation were aggravated.

**Diagnosis:**

Aspiration pneumonia (inhalation of nitric acid fumes), acute respiratory distress syndrome, and hypertension.

**Interventions:**

Veno-venous ECMO (VV-ECMO) was started 6 hours after exposure at the intensive care unit. During VV-ECMO, hypoxia improved. However, chest radiography revealed aggravated pulmonary edema. Prone positioning under ultrasound monitoring and high-dose methylprednisolone were administered on the first day. Nebulization and fiberoptic bronchoscopy for airway management were performed on the second day after the exposure. Pulmonary secretions were significantly reduced 48 hours later.

**Outcomes:**

The patient was weaned off V-V ECMO after 6 days, achieved the standard of extubation after 9 days, and was discharged without serious pulmonary or infectious complications after 12 days of hospitalization. Three weeks after discharge, the patient’s lung function showed a slight decline in the diffusion function. Two months after discharge, the patient’s lung function returned to normal.

**Lesson:**

Early ECMO combined with prone positioning and visualized management through ultrasonography can better improve the prognoses of patients and promote lung function recovery.

## 1. Introduction

Nitric acid is a strong oxidizing agent. Leakage of nitric acid occasionally occurs. The inhalation of nitric acid fumes can lead to toxic pulmonary edema. However, little evidence has been provided regarding the treatment of diseases caused by accidental inhalation of nitric acid fumes.^[1,2]^

Here, we report a healed case of nitric acid fume poisoning with veno-venous extracorporeal membrane oxygenation (VV-ECMO) treatment at an early time point after exposure and in a prone position (PP) during V-V ECMO treatment.

## 2. Case report

A 40-year-old man was admitted to the emergency department of our hospital because of accidental exposure to nitric acid fumes for 10 minutes in a factory. The industrial gas was composed of four volumes of 65% nitric acid. Mild throat irritation and dyspnea occurred 3.5 hours after exposure. Approximately 2 hours later, the severe dyspnea recurred. A noninvasive ventilator with breathing parameters (FiO_2_ 50%; Pi, 14; Ps, 6) was provided 12 hours after exposure. However, the oxygenation was not maintained. Tracheal intubation was performed 24 hours after exposure. However, even when high-level ventilator support was administered, the respiratory function deteriorated rapidly. Thus, VV-ECMO was used in the intensive care unit. The pre-ECMO survival probability was calculated using respiratory ECMO. The APACHE II score was 30.

The sequence of events for patients supported by V-V ECMO is outlined in the following paragraphs.

Exposure time (ET): Incident occurred. The patient was exposed to fumes of nitric acid for approximately 10 minutes. He had dry coughing and a feeling of suffocation for a very short period. At that time, no eye, nasal, or skin irritation was observed.

**Figure 1.**
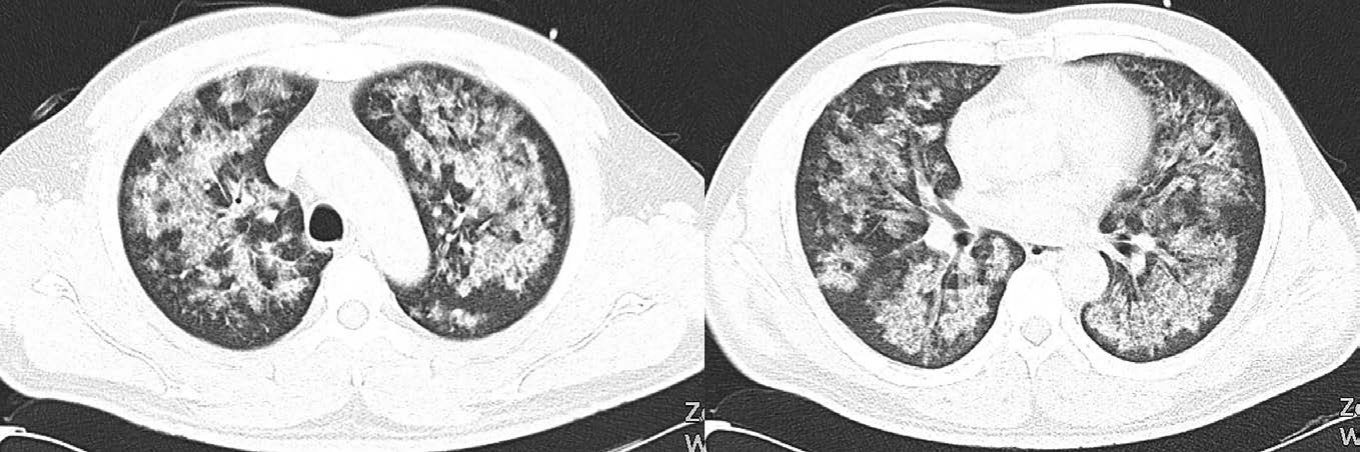
Typical image features at 4 hours after exposure time. Pulmonary edema could be observed on the computed tomography (CT) scan. Thoracic CT disclosed bilateral peribronchovascular ground glass opacity, as well as diffuse interstitial infiltrating shadow.

Four hours after ET, the patient experienced paroxysms of cough and shortness of breath. The patient was admitted to the emergency department of a local hospital. Chest computed tomography performed at a local hospital revealed bilateral interstitial edema (Fig. 1).

Twelve hours after ET, the patient was sent to the emergency department of our hospital with moderate-to-severe respiratory distress. Physical examination revealed a temperature of 38.5°C, respiratory rate of 45 breaths per minute, blood pressure of 145/85 mm Hg, and pulse of 136 beats/ min. Room air oxygen saturation was 80%. Noninvasive ventilation was initiated immediately.

24 hours after ET, the FiO_2_ of noninvasive ventilation was rapidly adjusted to 100% to maintain oxygen saturation. The arterial blood gas showed a pH of 7.32 (7.35–7.45), PaCO_2_ of 29 mm Hg (35–45 mm Hg), PaO_2_ of 56 mm Hg (75–100 mm Hg), and bicarbonate of 15 mmol/L (21–27 mmol/L), indicating that the patient was rapidly deteriorating into respiratory failure. The patient was intubated emergently, accompanied by continuous neuromuscular blockade. Methylprednisolone (200 mg) nebulized with bronchodilators, N-acetylcysteine, and sivelestat sodium were administered. Two hours after intubation, the patient’s respiratory condition worsened. Mechanical ventilation with positive end-expiratory pressure (PEEP) was used. Catecholamines were also administered (Norepinephrine 0.5 μg/kg/ min).

**Figure 2.**
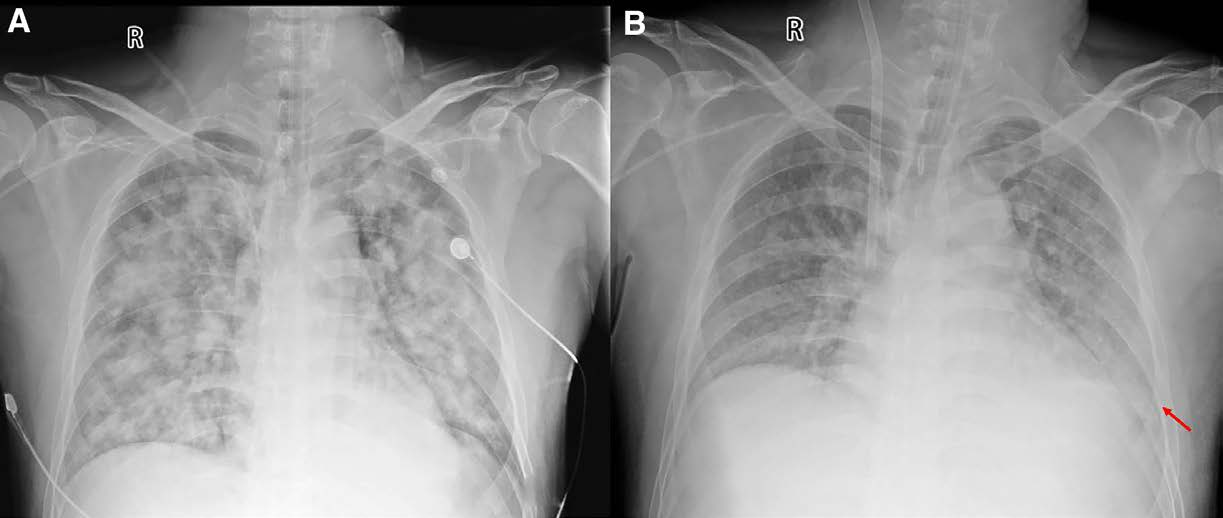
The chest X-rays at different times. (A) The X-ray on Day 1 after exposure showed the diffuse infiltration throughout all lung fields. (B) The X-ray on Day 5 after exposure showed that the diffuse plaques and infiltration in all lung fields were reduced obviously, and the costophrenic angle in the left region became blunt (red arrow).

30 hours after ET: Because of the persistent worsening of respiratory situation (pH 7.23, PaCO_2_ 78 cmH_2_O, PaO_2_ 52 cmH_2_O), inverse ratio ventilation (I:E 2:1, PEEP 10 cmH_2_O, FIO_2_ 1.0) was installed. Chest radiography revealed diffuse infiltration throughout all the lung fields (Fig. 2A). The decision was made to place the patient on a twin pulse extracorporeal life support system (BE-PLS: Maquet). A 17-F arterial and 21-F venous catheter were inserted into the femoral artery and vein percutaneously using a modified Seldinger method with visualization critical ultrasound. V-V ECMO was set to 2.5 a blood flow/min with an oxygen flow of 1.5 L/min. This resulted in the enhancement of oxygen for the next 6 hours and the continuous elimination of CO_2_. During the initial application of V-V ECMO, the blood pressure was 114/62 mm Hg and the heart rate was 92 bpm.

Subsequently, the patient required both V-V ECMO and PP for 12 hours per day for the next 4 days. A tracheal electronic endoscope was used to remove secretions and bronchoalveolar lavage fluid.

Day 5: The weaning process was started after successful improvement of lung function (FiO_2_ 0.3, PEEP 8 cmH_2_O, plateau pressure Pplat 17 cmH_2_O). Radiography showed that the diffuse plaques and infiltration in all lung fields were reduced, and the costophrenic angle in the left region was blunt (Fig. 2B).

Day 6: Extracorporeal blood flow was reduced stepwise to 1.5 L/min. The gas flow was tapered mostly parallel to the blood flow and finally shut off for 1 hours. The patient did not present with dyspnea or tachypnea. The V-V ECMO was successfully weaned off V-V ECMO.

Day 8: The mechanical ventilation was withdrawn.

Day 9: The patient was transferred to the respiratory department.

Day 11: The patient was discharged from the hospital. The laboratory results of patients at different time points are shown in Table 1.

**Table 1:** Laboratory results of the patient in different time points.

	D2 Pre-ECMO	D2 Post-ECMO	D3 Pre-prone position	D5 Post-prone position	D6 Wean off ECMO	D8 Wean off ventilator	D9 Transfer to respiratory department
PH	7.23	7.438	7.4	7.436	7.468	7.46	/
PaCO_2_	78	31	36.5	43.6	42.8	39	/
P/F	52/100	68/50	85/50	91/30	107/50	84	/
HCO_3_-		22	22.6	29	31	29	/
SaO_2_		96	97	97	96	97	/
ScvO_2_		61	63	76	81	/	/
Lactate		2	2.11	1.96	2.1	2.16	/
Hemoglobin		143	129	88	98	115	131
WBC		21	21.68	10.34	15	15.41	15.51
Na+		144	142.5	150	143	135.5	140
K+		4.4	4.12	4.2	4.1	3.86	3.9
Bun/Cr		/	11.08/145.08	8.4/81	7.36/64.3	7.9/69	7.9/69
ECMO air	/	4	4	1	/	/	/
ECMO Blood	/	3.8	4.5	2.5	/	/	/
MV (FiO2) %		50	50	30	50	/	/
Cdyn		29	22	49	/	/	/
Pplat		22	24	17	/	/	/
PEEP	10	10	12	8	8	/	/

Two months after discharge, the patient’s lung function returned to normal (Table 2).

**Table 2:** Results of lung function tests.

	3weeks postexposure	8weeks postexposure		
		% predicted		% predicted
FEV_1_ (L)	3.13	89	3.23	92
FVC (L)	3.77	82	3.75	82
FEV_1_/FVC (%)	83	108	86	113
TLC (L)	5.5	91	6.08	101
RV (L)	1.73	97	1.95	109
DLCO (mL/mm Hg/min)	19.52	77	30.48	119
RAW (cmH_2_O/L/s)	0.4	135	1.65	114

## 3. Discussion

Inhalation of nitric acid fumes leads to toxic pulmonary edema, which can be caused by strong stimulation of and corrosive effects on lung tissue.^[3]^ The fumes can damage the surfactant substances and capillary wall permeability of the lung, resulting in the enhancement of alveolar wall permeability, which could cause the body fluids in the capillaries to permeate into the lung interstitium and alveoli.^[4]^ In addition, the excitement of synthetic nerves can cause spasms of the right total lymphatic tube, resulting in a disordered lymphatic reflux. Subsequently, acute lung injury occurs.^[2]^

Based on a search of PubMed in June 2021, we reviewed all papers using the terms ‘nitric acid fume inhalation, nitric acid inhalation AND accident, nitric acid inhalation, nitric acid inhalation AND toxic, nitric acid AND corticosteroids,’and ‘nitric acid AND ECMO’. Based on the references in these articles, we found additional papers on these topics. Sixteen cases of inhalation injury to nitric acid fumes were reported in ten articles (Table 3).^[1,3–11]^ Fourteen patients’ exposures were related to industrial work. Two other cases were exposed during farmer work and leisure cleaning. In industrial work-related exposures, four patients were intoxicated during tank cleaning activities performed without respiratory protection. There were 6 severe acute respiratory distress syndrome (ARDS) cases among the 16 exposed patients. Of the 6 patients with severe ARDS, four died. ECMO was used in two surviving patients.

**Table 3:** Literature review of cases with inhalation injury of nitric acid.

Literature	Author and Publish years	Patient number (N)	Gender and age	Inhalation concentration, contact form and ET	Clinical manifestations	ARDS classification	Treatment	Outcomes
1	Hajela et al;1990^[3]^	3	Males;36/44/21 y	NA: 68%;Fumes spill;ET:15 min	4–6 h AE: all increasing dyspnea.7–9 h AE: cyanotic with frothy fluid escaping from the nose and mouth. R: 28–44; HR: 100–168; PO_2_ 37-58 (FiO_2_ nm); X-ray: diffuse alveolar pattern and pulmonary edema	Severe	One of them was intubated at 7 h AE, the others were intubated at 9 h AE	All 3 patients died within 24 h AE
2	Andreas Bur et al;1997^[10]^	1	Male;56 y	NA: 68%;Fumes spill;ET:15 min	30 min AE: respiratory distress.1 h AE: BP: 120/60 (vasoactive agents); HR: 100; X-ray: pulmonary edema; P/F: 98	Severe	Intubated at 3 h AE, and treated with ECMO at 8 h AE	The complication of ECMO: the right leg became ischemic.The patient died from refractory respiratory failure at the 4th day AE
3	Shin et al;2007^[1]^	2	Males;37/43 y	NA: 65%, HF: 55%;Fumes spill;ET: 5 min	2 h AE: BP: 86/55, 140/74; HR: 116, 134; X-ray: severe pulmonary edema; P/F: 51–54	Severe	Both were intubated.One of them was treated with ECMO at 7 h AE, and the N- acetylcysteine was also used	One died at 3.5 h AE. Another was rescue with the treatment of ECMO for 8 d, and discharged at the 18th day AE
4	Kao et al;2008^[11]^	2	Males;27/32 y	NA: 68%;Fumes spill;ET: 10 min	AE: cough and vomiting soon.12 h at ED: dyspnea, RR: 24–30, X-ray: bilateral shadowing; P/F: 285	Mild	One of them was treated with non-invasive ventilator (weaned off after 12 h). Another was treated with high-flow oxygen	Both were discharged at the 5th day AE.
5	Murphy et al;2008^[5]^	1	Male;66 y	NA: 70%;Tank cleaning;ET: 45 min	AE: None.4 h AE: dyspnea at ED, RR: 8; SpO_2_ 97% on room air; BP and HR: nl.5 h AE: pulmonary edema, P/F: 74 on mask at 100% O_2_	Severe	Intubated at 10 h AE	Died at 53 h AE due to hemodynamic and respiratory decompensation
6	Jayalakshmi et al;2009^[6]^	3	Males;30/35/28 y	NA: nm;Tank cleaning;ET: 10 min	All of them appeared with dyspnea and dry cough. One of them had mild hypoxia. The other two: RR: 40–44; SpO_2_ 88% with high flow oxygen mask	Mild	Only one was intubated and weaned off after 4 d. The patient was treated with methylprednisolone, antibiotics and nebulized with bronchodilators and N-acetylcysteine	All 3 men discharged at 7th day AE
7	Lee et al;2012^[7]^	1	Male;Nm	NA: nm;Electroplating;ET: 5 min	AE: mild throat dyspnea.2.5 h AE: cyanosis and frothy secretion. RR: 28; BP: 140/74; HR: 134; X-ray: diffuse interstitial infiltrates and ground glass opacities in both lungs. P/F: 43.7.	Severe	Intubated at 4 h AE and weaned from ECMO at the 7th day	Discharged at 3rd week AE
8	Lee et al;2014^[4]^	1	Male;50 y	NA: nm;Tank cleaning;ET: 4 h	AE: none. First symptom was coughing several days afterwards. Crackles were found in both lung bases. CT: interstitial and peribranchial ticking with early bronchiectasis	Mild	VATS biopsy: BOOP.Prednisolone for 8 months, and reduced to 5 mg daily for another month	Normal lung function after 9 mo
9	Kido et al;2017^[8]^	1	Male;50 y	NA: nm;Electroless nickel plating;ET: nm	AE: coughing and shortness of breath.14 h AE: R: 26; non-rebreather mask with 6L/min oxygen and SpO_2_ reached to 96%; HR: 86; PO_2_: 139	Mild	Methylprednisolone pulse therapy (500 mg/d intravenously for 3 d), gradually reduced for 30 d, supplemental oxygen by nasal cannula	Normal lung function after 9 d, and discharged at the 15th day AE
10	Meaden et al;2019^[9]^	1	Male;49 y	NA: nm;Fumes spill;ET: 6 h	AE: none.12 h AE: paroxysms of coughing and shortness of breath; RR: 34; BP: nl; P: 87; ECG: nl; SpO_2_: 92% (FiO_2_: 40%); X-Ray: bilateral pulmonary infiltrates and pulmonary edema, P/F: 146	Moderate	Supplemental oxygen by nasal cannula, and treated with bronchodilator	Discharge with nl lung function one month later

Shin et al reported that patients who were treated early with ECMO combined with N-acetylcysteine after nitric acid inhalation were weaned from ECMO after 8 days and discharged normally after 18 days in 2007.^[1]^ Lee et al reported a patient who was weaned from ECMO for 7 days and discharged without complications in 2014.^[4]^ In our case, lung compliance and oxygenation were significantly improved on the fifth day, ECMO was withdrawn at 7 days, and the patient was discharged successfully on the 12th day. The pulmonary function was slightly limited. Two months after discharge, the patient’s lung function returned to normal. The use of early extracorporeal life support before the aggravation of tissue hypoxia is very important in patients with inhalation lung injury.^[12]^

Some studies have suggested that PP treatment with VV-ECMO support should be considered in these patients.^[13,14]^ PP can not only can promote sputum drainage, especially in chemical inhalation pneumonia, but also can avoid gravity dependent zone atelectasis. By improving oxygenation, hemodynamics can expedite weaning.^[15,16]^ The use of PP is an important reason for the rapid withdrawal of ECMO in this patient. This phenomenon has not been reported previously.

Another important method in the treatment of the patient was visualized management using ultrasound. Critical ultrasound has transformed ECMO from technology to precise management.^[17,18]^ Extracorporeal Life Support Organization showed that ultrasound was mandatory during the initiation of ECMO. Cannula insertion, hemodynamics, monitoring, and detection of complications can be assisted with ultrasound to shorten the operation time of ECMO.^[19]^ Left room pressure, monitoring of lung B-line conditions, and dynamic adjustment of patient capacity were assisted by ultrasound. In our case, the chest radiograph showed rapid improvement in pulmonary edema, depending on dynamic cardiopulmonary ultrasound assessment. We recommend visualizing critical ultrasound intervention in the management of all ECMO patients to avoid inappropriate interventions and achieve better implementation of precision treatment.

## 4. Conclusion

Severe ARDS caused by nitric acid inhalation is rarely associated with inhalation pneumonia. Early ECMO combined with PP and visualized management through ultrasonography can improve the prognosis of patients and promote lung function recovery.

## Acknowledgments

We thank Dr. Wanhong Yin and Dr. Wei Lai from West China Hospital of Sichuan University for their technical assistance.

## Author contributions

Conceptualization: Qian Wang.

Funding acquisition: Qian Wang.

Project administration: Lvlin Chen, Qian Wang.

Resources: Junchen Zhu, Yan He, Chao Huang.

Supervision: Ying Lan, Liyuan Peng.

Writing – original draft: Qian Wang, Hui Li.

Writing – review & editing: Qian Wang, Hui Li.
